# The Activity of Ten Natural Extracts Combined in a Unique Blend to Maintain Cholesterol Homeostasis—In Vitro Model

**DOI:** 10.3390/ijms23073805

**Published:** 2022-03-30

**Authors:** Sara Ruga, Rebecca Galla, Claudia Penna, Claudio Molinari, Francesca Uberti

**Affiliations:** 1 Laboratory of Physiology, Department of Translational Medicine, University of Piemonte Orientale, Via Solaroli 17, 28100 Novara, Italy; sara.ruga@uniupo.it (S.R.); rebecca.galla@uniupo.it (R.G.); 2 Laboratory of Cardiovascular Physiology, Dipartimento di Scienze Cliniche e Biologiche, Università degli Studi di Torino, Regione Gonzole 10, 10043 Orbassano, Italy; claudia.penna@unito.it; 3 Dipartimento per lo Sviluppo Sostenibile e la Transizione Ecologica, University of Piemonte Orientale, 13100 Vercelli, Italy; claudio.molinari@uniupo.it

**Keywords:** cholesterol homeostasis, cardiovascular disease, low-density lipoprotein, statin, monacolin K, food supplement, nutraceuticals

## Abstract

Background: Hypercholesterolemia is a major cause of cardiovascular disease and statins, the HMGCoA inhibitors, are the most prescribed drugs. Statins reduce the production of hepatic cholesterol, leading to greater expression of the LDL receptor and greater absorption of circulating LDL, reducing peripheral LDL levels. Unfortunately, statins are believed to induce myopathy and other severe diseases. To overcome this problem, safe nutraceuticals with the same activity as statins could hold great promise in the prevention and treatment of hypercholesterolemia. In this study, the anti-cholesterol efficacy of a new nutraceutical, called Esterol10^®^, was evaluated. Methods: HepG2 cells were used to study the biological mechanisms exerted by Esterol10^®^ analyzing different processes involved in cholesterol metabolism, also comparing data with Atorvastatin. Results: Our results indicate that Esterol10^®^ leads to a reduction in total hepatocyte cholesterol and an improvement in the biosynthesis of free cholesterol and bile acids. Furthermore, the anti-cholesterol activity of Esterol10^®^ was also confirmed by the modulation of the LDL receptor and by the accumulation of lipids, as well as by the main intracellular pathways involved in the metabolism of cholesterol. Conclusions: Esterol10^®^ is safe and effective with anti-cholesterol activity, potentially providing an alternative therapy to those based on statins for hypercholesterolemia disease.

## 1. Introduction

As is well known, a high blood cholesterol level is the 6th highest risk factor for death worldwide [1]. Several factors including diets high in saturated fat, physical inactivity, and genetic factors can raise cholesterol levels in humans. This is the main risk of heart disease, stroke, and other vascular diseases. Individuals with elevated low-density lipoprotein cholesterol (LDL-C) and high levels of total cholesterol [2] are prone to the development of coronary heart disease through numerous pathogenetic mechanisms. Nutraceuticals are a class of products on the border between nutrients and drugs that provide supplementation of specific nutrients with beneficial effects on health [1]. The lipid-lowering nutraceuticals currently available are varied in terms of the mechanism of action and effectiveness in reducing the cholesterol level [3]. Among these, those derived from plants are considered the best for improving the plasma lipid profile because they mainly act in reducing the levels of LDL-C which is the main approach for managing coronary heart disease (CHD) and cardiovascular (CVD) risks [4].

Several statins are commercially available, and their use can be selected based on the patient’s individual needs, therapeutic goals, and tolerability [5]. Surely, statins are recommended for dyslipidemia to reduce the risk of primary and secondary cardiovascular disease as they play an essential role in lowering LDL-C cholesterol levels, as well as having other non-lipid properties, such as inflammatory, antithrombotic, antioxidant, or antiapoptotic activity [6]. Furthermore, statin-based therapy may not be sufficient to reduce cardiovascular risk in high and very high-risk patients [5]. For this reason, research has been dedicated to the study of different natural compounds. Indeed, herbal remedies are increasingly used in an attempt to improve the lipid profile of individuals with early atherosclerosis and those with other risk factors, such as hypertension or diabetes mellitus [7]. As a result, innovative nutritional strategies have been developed to reduce major cardiovascular risk factors, including dietary changes or consumption of functional foods and dietary supplements specifically targeted for the treatment of dyslipidemia. Nowadays, there is a great need to determine a recommendation for the possible role of nutraceuticals in patients with statin-associated adverse effects. However, at the same time, it is fundamental to underline that the nutraceuticals, both in patients with good adherence to statin therapy and with statin intolerance, cannot replace drug therapy, but could help achieve therapeutic goals [6]. In this context, it is important to consider the available clinical evidence on the efficacy and safety of fermented red rice extract (RYR), a very popular natural extract with some lipid-lowering effects. RYR is obtained from the fermentation of yeasts (e.g., *Monascus purpureus*, *M. pilosus*, *M. floridanus*, *M. ruber*, and, more recently, *Pleurotus ostreatus*) in red rice (Oryza sativa) which is the reason for the typical red color. This process produces a complex of substances called monacolins which are now widely accepted as lipid-lowering molecules. The concentration of monacolins in the most commonly used RYR-containing nutraceuticals usually reaches up to 1.9%. Several types of monacolins have been identified and one of these subtypes is monacolin K, which was first isolated from Akira Endo and found to be structurally identical to lovastatin. Its main mechanism of action is to inhibit 3-hydroxy-3-methyl-glutaryl-CoA (HMGCoA) reductase, the enzyme that controls the speed of the cholesterol synthesis pathway. Although monacolin K and lovastatin have the same structure, their pharmacokinetic profiles and bioavailability are different. In fact, monacolin K is only one of several RYR components that can modify the pharmacokinetic profile of lovastatin [2]. However, several adverse effects associated with the intake of RYR products have been reported during clinical trials in a similar way to that seen from treatment with lovastatin. The World Health Organization (WHO) Vigibase database from 2002 to 2018 reported 82 cases of adverse effects to RYR mainly related to musculoskeletal and connective tissue disorders (39%), general disorders and administration site conditions (32.9%), and gastrointestinal disorders (18%). In particular, between April 2002 and September 2015, the Italian Surveillance System of Natural Health Products collected 52 reports (3.7 cases/year) relating to 55 adverse events in 14 years related to the consumption of RYR, of which three were associated with a monacolin K dose equal to 3 mg/day. Adverse events (164 cases) following RYR consumption were also reported by the US Food and Drug Administration (FDA) from 2004 to 2017 [5]. Some meta-analyses showed that the effect of RYR on LDL reduction was no different from moderate-intensity statins (0.03 mmol/L) such as pravastatin 40 mg and lovastatin 20 mg. Indeed, the authors found a small increase in HDL-associated cholesterol (HDL-C) and a decrease in triglycerides (TG), and an improvement in endothelial function. Additionally, the authors documented the possible synergistic effects of RYR with other antioxidants such as green tea dry extract, Coenzyme Q10 (CoQ10), and resveratrol [2]. In particular, the interaction between RYR and natural extracts with different mechanisms of action can have a synergistic effect and the study highlighted that the intake of RYR can be combined with other botanicals or other drugs, which can prevent possible side effects [5]. Several studies have shown that natural extracts containing antioxidants, or in any case able to counteract oxidative stress, can represent a promising adjuvant therapy in hypercholesterolemia. Indeed, these molecules are able to reduce the risk of cardiovascular diseases by increasing the resistance to oxidation of LDL-C [8]. Furthermore, AMP-activated protein kinase (AMPK) has recently been evaluated as a key regulator of a naturally extracted active ingredient capable of preventing CVD. For example, it is suggested that polyphenolic compounds can be used to activate AMPK to reduce oxidative damage and inflammation, which play a critical role in the development of chronic disease [9]. In nature, several nutraceutical combinations have shown significant lipid-lowering effects and a potential positive impact on cardiovascular risk by modulating the proprotein convertase subtilisin/kexin type 9 (PCSK9) factor which plays a particular role in regulating LDL-C levels by degrading the LDL receptor (LDLr) [10]. Based on all these results it is important to resolve the claim about some nutraceuticals with RYR by studying the combined effects of several active ingredients, such as Sage extract, Resveratrol, Folic Acid, vitamin D3, Quercetin, Astaxanthin, and CoQ10. Sage extract is still used in traditional medicines for its hypoglycemic action [11], and it has antioxidant properties due to its phenolic nature [11,12]. The utility of this extract is demonstrated by the significant increase in the hepatic antioxidant glutathione-S-transferase enzyme observed both in vitro and in vivo [13]. Resveratrol is another extract that is able to maintain homeostasis of lipid balance [14,15,16], as well as folic acid supplementation which in recent years has increased its role in CVD, as reported in the literature. Dietary supplementation with folic acid has been shown to induce several positive effects against, for example, cholesterol accumulation [17].

Several studies indicate that in subjects with vitamin D3 deficiency, plasma high-density lipoprotein (HDL) levels are reduced, promoting the hypercholesterolemic condition. Consequently, supplementation with vitamin D3 works by controlling the outflow of cholesterol [18]. Quercetin also appears to be able to significantly reduce cholesterol concentration by regulating both LDLr and HMGCoA [19]. Furthermore, astaxanthin appears to be able to inhibit lipogenesis and lipid deposition by decreasing intracellular TG due to its antioxidant properties and can be of great use, [20] as well as CoQ10, which could prevent the occurrence and progression of metabolic diseases in a similar manner [21]. Finally, it is also advisable to consider Vitamin K2 which can redirect the accumulation of lipids towards a safe fraction of triacylglycerols [22], especially when combined with sodium selenite to influence cholesterol metabolism by affecting the reduced glutathione/oxidized glutathione (GS/GSSG) ratio and HMGCR (3-hydroxy-3-methyl-glutaryl-coenzyme A reductase) [23]. However, the cholesterol-fighting ability of a combination of all these substances associated with RYR has not yet been explored in liver tissue. The liver is a multifunctional organ that deals with the regulation of many critical processes and is responsible for the homeostasis of lipids, regulating their absorption, distribution, and storage, β-oxidation, and lipogenesis. The progressive accumulation of fat in the liver is one of the main processes responsible for impaired liver function. The liver is also the main site of cholesterol homeostasis and is the main target of numerous therapies, including the use (and abuse) of statins to combat the condition of hypercholesterolemia. To perform a preliminary screening of the plant extracts and the efficacy and safety of the formulation, an in vitro approach using a usually preferable cell model of proliferating human hepatoma, HepG2 cells was selected.

The HepG2 cell line derived from human hepatoblastoma has many functions attributed to a normal human hepatocyte, including a canalicular region of bile, very similar to the morphology of the primary hepatocyte. As reported above, this cell line secretes various plasma proteins, including apolipoproteins, and can synthesize and secrete lipoproteins (from very-low density lipoprotein, VLDL, to HDL). Furthermore, these cells maintain the ability to express the main enzymes that regulate the metabolism of hepatic, plasma, and biliary cholesterol and appear to be able to synthesize and secrete bile acids. These activities respond consistently to what is known about human cholesterol metabolism [24]. For this reason, this cellular model was applied in this study to verify the safety and efficacy of a new combination of various natural ingredients with RYR in order to create the basis for a new therapeutic strategy for humans.

## 2. Results

### 2.1. Effects of Several Natural Extracts, RYR, and Atorvastatin on HepG2 Cell Viability

**Figure 1:**
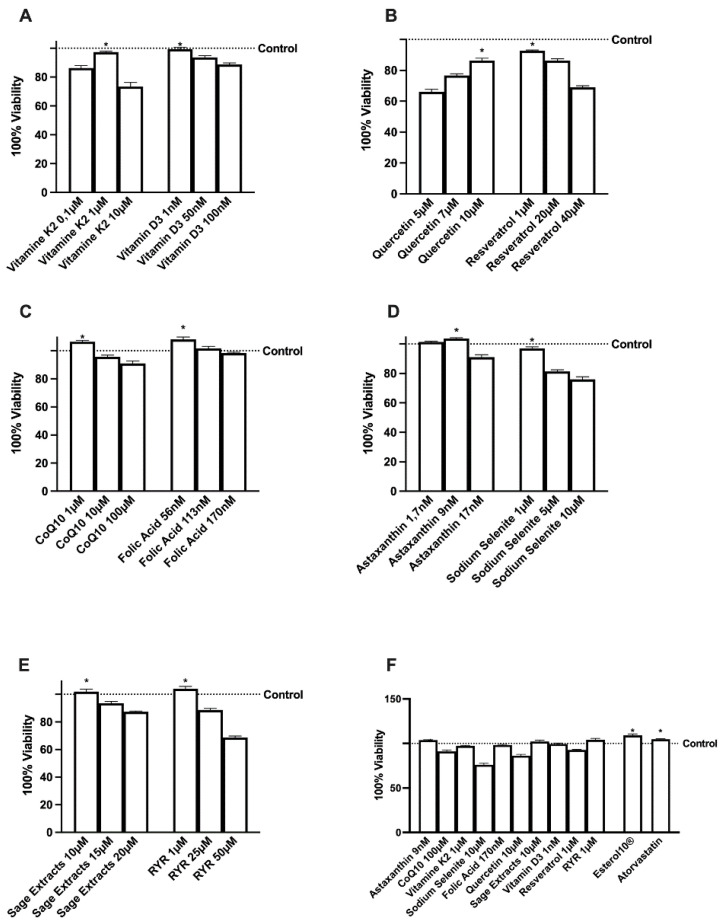
Cell viability of HepG2 cells incubated with the agents alone and combined, for 24 h. RYR = Red Yeast Rice. From A to E the dose-response study on cell viability was measured by MTT test of each single extract such as vitamin K2 and vitamin D3 (**A**), quercetin and resveratrol (**B**), CoQ10 and folic acid (**C**), astaxanthin and sodium selenite (**D**), sage extract and RYR (**E**). In (**F**) the effects of the better concentration of single agents alone and combined (Esterlo10^®^) were compared to RYR and atorvastatin alone. Viability was calculated as the reduction percentage of cells in the culture medium without the addition of test substances. Data are mean ± SD of five independent experiments performed in triplicates. * *p* < 0.05 vs. control.

Before exploring the potential anti-cholesterol activity of the new formulation, the effects of the single agents and the RYR alone were evaluated on the viability of HepG2 cells by means of a dose-response study to verify the toxicological analysis, considering 24 h as the relevant exposure time. As reported in Figure 1A–E, all substances were able to induce a reduction in HepG2 cell viability as well as reported in the literature, compared to control. These molecular mechanisms may underlie the agent-induced apoptosis or self-protective response of HepG2 cells. For this reason, the concentration able to maintain or produce little increase in cell viability (*p* > 0.05 vs. control) was chosen for each agent to be used in the final formulation (Figure 1F). In particular, Sage extract 10 µM, Quercetin 10 µM, Vitamin D3 1 nM, Vitamin K2 1 µM, CoQ10 1 µM, Resveratrol 1 µM, Astaxanthin 9 nM, Sodium Selenite 1 µM, Folic Acid 1 µM, and RYR 1 µM were chosen. In addition, when all the agents were added together, no significant changes were observed indicating maintenance of a self-protection response even by the complete formulation (named Esterol10^®^). Finally, no adverse effect on HepG2 cell viability was observed for atorvastatin.

### 2.2. Effects of Esterol10, RYR, and Atorvastatin on Hepatic Cholesterol Biosynthesis

**Figure 2:**
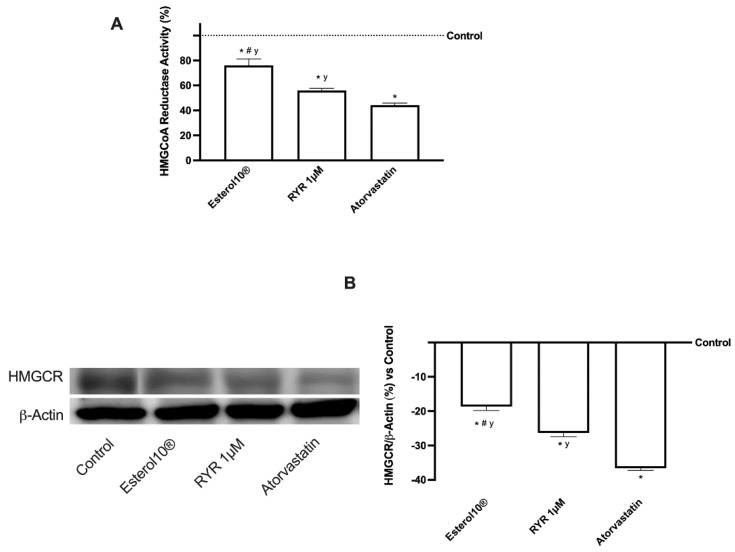
HMGCoA reductase activity (**A**) and HMGCR expression (**B**) on HepG2 cells. The abbreviations are the same reported in Figure 1. Data are mean ± SD of five independent experiments performed in triplicates. In panel B, the images reported are an example of a Western blot obtained with technical replicates. * *p* < 0.05 vs. control; ^#^ *p* < 0.05 vs. RYR; ^y^ *p* < 0.05 vs. Atorvastatin.

In light of the results obtained, the evaluation of anti-cholesterol activity was investigated by testing Sage extract 10 µM, Quercetin 10 µM, Vitamin D3 1 nM, Vitamin K2 1 µM, CoQ10 1 µM, Resveratrol 1 µM, Astaxanthin 9 nM, Sodium Selenite 1 µM, Folic Acid 1 µM, and RYR 1 µM in a unique blend (named as Esterol10^®^) and comparing the data with RYR 1 µM alone and to Atorvastatin 1.25 µM. The biosynthesis of cholesterol was evaluated by analyzing HMGCoA reductase, absorption through LDLr and quantification of lipoproteins, accumulation by total and free cholesterol, and conversion into bile acids following the physiological homeostasis. As reported in Figure 2, HMGCoA reductase that catalyzes the production of mevalonate from HMGCoA which is the rate-limiting step for cholesterol synthesis was analyzed by ELISA (Figure 2A and Table A1) and Western blot and the relative densitometric analysis (Figure 2B) on HepG2 cells treated with Esterol10^®^ was compared to data from RYR and Atorvastatin. Esterol10^®^ was able to statistically reduce the activity of the enzyme and the receptor expression compared to RYR (about 35% and about 30%, respectively) and Atorvastatin (about 72% and about 50%, respectively), supporting the ability of the combination to prevent the negative consequences of the chronic use of statins leading to the diabetogenic effects. Furthermore, the RYR revealed fewer adverse effects similar to atorvastatin (approximately 27% and approximately 28%, respectively), supporting the hypothesis on the concentration of RYR-dependent side effects.

### 2.3. Effects of Esterol®, RYR, and Atorvastatin on Cholesterol Metabolism

**Figure 3:**
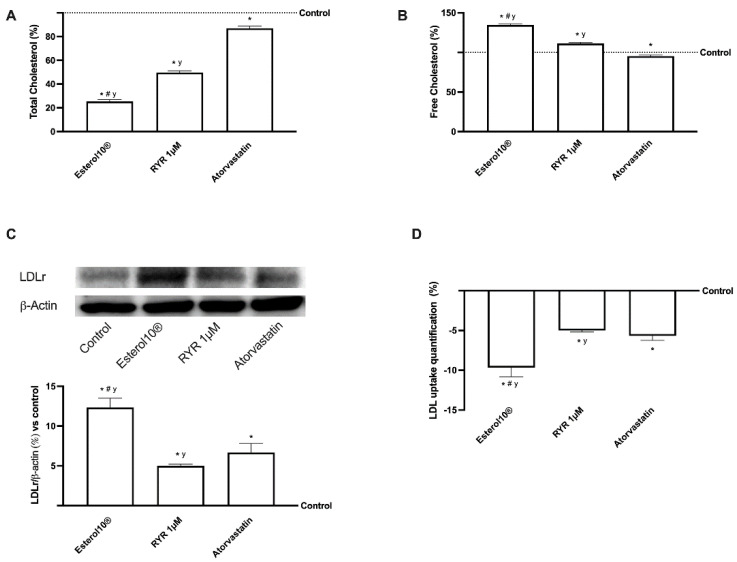
Total Cholesterol (**A**), Free Cholesterol (**B**), LDLr (**C**), and LDL uptake quantification (**D**) on HepG2 cells. The abbreviations are the same reported in Figure 1. Data are mean ± SD of five independent experiments performed in triplicate. In (**C**), the images reported are an example of a Western blot obtained with technical replicates. * *p* < 0.05 vs. control; ^#^ *p* < 0.05 vs. RYR; ^y^ *p* < 0.05 vs. Atorvastatin.

Since the hepatic cholesterol after the enzymatic esterification and incorporation into lipoproteins is transported to the peripheral tissues by LDL or excreted as free cholesterol or eliminated by cholesterol-derived bile acids, additional experiments were carried out to analyze this mechanism. Consequently, it is important to define if Esterol10^®^ is an effective treatment for hypercholesterolemia compared to 1 µM RYR and 1.25 µM Atorvastatin by reducing cholesterol through lowering total cholesterol and/or by increasing free cholesterol hepatic content. As shown in Figure 3A, all formulations tested are able to significantly reduce the total cholesterol content in HepG2 cells (*p* < 0.05 vs. control by all substances); in particular Esterol10^®^ exerted the main effect compared to RYR (about 56%) and Atorvastatin (about 74%) supporting the better influence of the single agents combined in the formulation. In addition, only Esterol10^®^ is effective in increasing free cholesterol production (Figure 3B); in particular, this effect was more evident compared to RYR (*p* < 0.05, about 22%) which induced a little increase compared to control (*p* < 0.05). As expected, atorvastatin showed no effect on free cholesterol, while total cholesterol was significantly reduced by approximately 20%.

To confirm the anti-cholesterol activity, the involvement of the LDLr and the consequences of LDL uptake on HepG2 cells were also investigated by Western blot and ELISA. In fact, by increasing the expression of the LDLr gene, the LDLr protein on the plasma membrane of the hepatocytes and the absorption of LDL were improved by Esterol10^®^ (*p* < 0.05) compared to RYR (about 1.5 times more on the LDL receptor and about 90% LDL uptake) and atorvastatin (about 1 time over LDL receptor and about 80% LDL uptake) as shown in Figure 3C,D.

### 2.4. Effects of Esterol10^®^, RYR, and Atorvastatin on Choleresis Process

**Figure 4:**
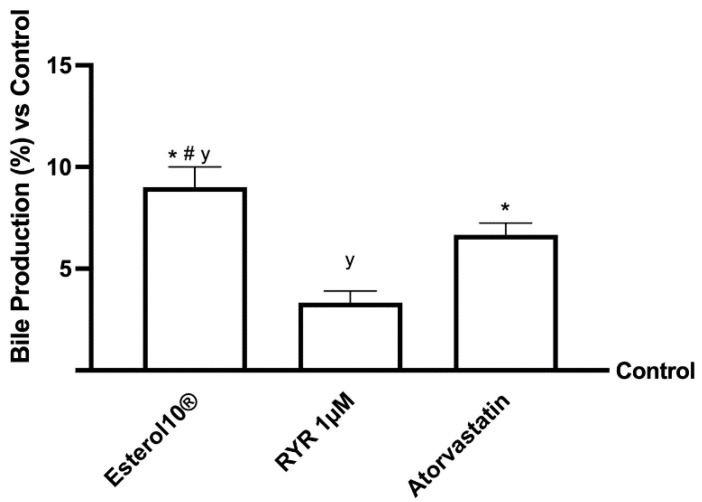
Bile acid production following treatment of hepatic in vitro model. The abbreviations are the same as reported in Figure 1. Data are mean ± SD of five independent experiments performed in triplicate. * *p* < 0.05 vs. control; ^#^ *p* <0.05 vs. RYR and ^y^ *p* < 0.05 vs. Atorvastatin.

Increased hepatic free cholesterol content is the major form of cholesterol released through bile, and is a key step in general cholesterol homeostasis (route of excretion). As the control of this production is considered an important target for the treatment of hypercholesterolemia, the potential ability of 1 µM of Esterol10, 1 µM RYR, and 1.25 µM Atorvastatin to increase the conversion of cholesterol to bile acid was therefore investigated (Figure 4). In particular, Esterol10^®^ has been shown to significantly increase bile acid production in the in vitro hepatic model by approximately 1.5-fold more than RYR (*p* < 0.05) and one-fold more than Atorvastatin (*p* < 0.05), indicating that it is the best formulation with choleretic action. Furthermore, the RYR increased bile acid production by approximately 9% compared to the control, while atorvastatin was able to increase bile acid production by approximately 6%, less than the RYR, confirming their ability to stimulate the synthesis of bile acids.

### 2.5. Effects of Esterol10^®^, RYR, and Atorvastatin on the Intracellular Pathways Leading to Cholesterol Homeostasis

**Figure 5:**
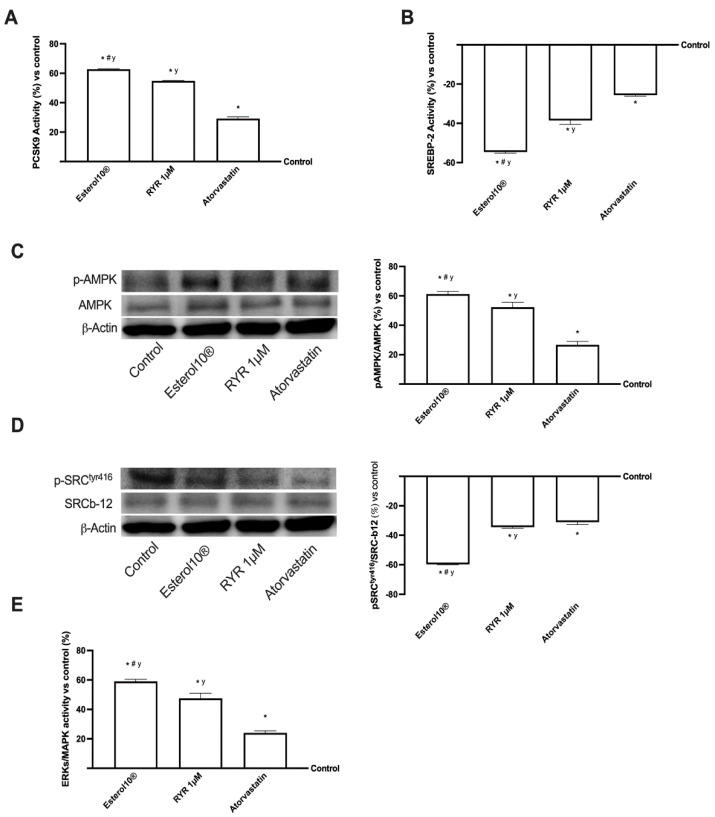
Intracellular pathways activated in HepG2 cells. In (**A**,**B**,**E**) the protein activity of PCSK9, SRC, and ERK was measured by an ELISA test; in (**C**,**D**) the analysis of AMPK and SRC performed by Western Blot and densitometric analysis. The abbreviations are the same reported in Figure 1. Data are mean ± SD of five independent experiments performed in triplicates. The images reported are an example of a Western blot obtained with technical replicates. * *p* < 0.05 vs. control; ^#^ *p* < 0.05 vs. RYR; ^y^ *p* < 0.05 vs. Atorvastatin.

Many nutraceuticals and statins have also been shown to induce the expression of the PCSK9 gene which binds directly to LDLr to lower plasma cholesterol. This mechanism may be involved in the activation of the transcriptional activity of the sterol responsive element binding protein (SREBP). In particular, SREBP-2 preferentially transcribes the genes involved in the biosynthetic pathway of cholesterol. This mechanism is also important for activating the AMPK/SREBP pathway to reduce lipid accumulation in the liver. AMPK is an important protein involved in energy metabolism and the regulation of fatty acid metabolism [9,25]. In this context, as reported in Figure 5, these proteins and their mechanisms have also been studied in HepG2 cells after stimulation with 1 µM Esterol10^®^, 1 µM RYR, and 1.25 µM Atorvastatin. As shown in panel A, B and Table A1, Esterol10^®^ is able to improve the activity of PCSK9 (about 20% more than 1 µM RYR and about 2 times more than Atorvastatin, *p* < 0.05); on the contrary, treatment with Esterol10^®^ inhibits the activity of SREBP-2, about 42% less than 1 µM RYR and about once more than Atorvastatin (*p* < 0.05), showing that SREBP phosphorylation can regulate protein expression, and confirming the involvement of AMPK/SREBP signaling in treatment with Esterol10^®^. Since PCSK9 is also directly associated with CD36, which is often associated with inflammation and steatosis, it regulates LDLr levels and complex the AMPK signaling cascade which includes the SRC kinase [26]. Therefore, the same trend observed by PCSK9/SREBP, was also observed by the expressions AMPK (panel C) and SRC (panel D). Indeed, Esterol10^®^ is able to activate the AMPK pathway (about 18% more than 1 µM RYR and about 90% compared to Atorvastatin, *p* < 0.05) and to inhibit SRC (about 73% less than 1 µM RYR and about 91% compared to Atorvastatin, *p* < 0.05) indicating that Esterol10^®^ plays a fundamental role on the AMPK/SRC pathway supporting our hypothesis of an active role of Esterol10^®^ in the modulation of cholesterol metabolism. Furthermore, numerous evidence indicates that the extracellular signal-regulated kinases (ERK) signaling pathway is involved in the regulation of PCSK9 and LDLr [27]; thus, to further confirm the activity of Esterol10^®^ in regulating the cholesterol level, we also studied the activity of ERKs/MAPK by means of ELISA tests. As shown in panel E and Table A1, Esterol10^®^ could increase the ERK1/2 signal pathway (approximately 25% compared to 1 µM RYR and approximately 1.5 times more than Atorvastatin, *p* < 0.05) indicating, once again, which could regulate cholesterol metabolism even at the beginning of the cascade signaling. Collectively, these results suggest that Esterol10^®^ enhanced PCSK9 expression and reduced SREBP-2 activity in HepG2 cells, also regulating the AMPK/SRC pathway through activation of the ERK1/2 signaling pathway.

### 2.6. Effects of Esterol10^®^, RYR, and Atorvastatin on Liver Injury Markers

**Figure 6:**
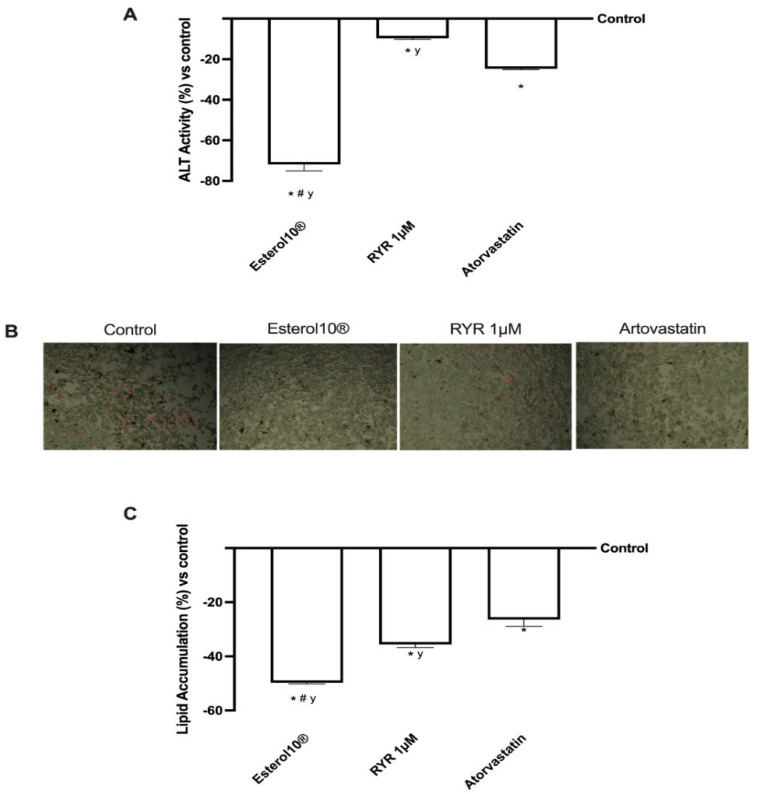
Tissue integrity analysis. In (**A**), ALT activity was measured by an ELISA test. Images captured under the microscope at an original magnification of ×20 and reported in (**B**) showed the intercellular oil droplets stained by the Oil Red stain. These oil droplets are solubilized for quantification by spectrophotometric analysis reported in (**C**). The abbreviations are the same as shown in Figure 1. Data are mean ± SD of five independent experiments performed in triplicate. * *p* < 0.05 compared to the control; ^#^ *p* <0.05 vs. RYR; ^y^ *p* <0.05 vs. Atorvastatin.

Some researchers have suggested that statin and monacolin K treatment is closely related to biochemical parameters responsible for liver injury. To explore this aspect, the tissue damage associated with our treatment was assessed by measuring alanine aminotransferase (ALT). According to the results shown in Figure 6 (panel A), ALT activity decreased significantly after treatment with Esterol10^®^ compared to the control (*p* < 0.05) and compared to 1 µM RYR (about 7 times more) and to Atorvastatin (about 2 times more) confirming the positive role of Esterol10^®^ in reducing liver damage; this is normally found after chronic treatment with statins, which as known leads to other risk factors, such as hypertension or diabetes mellitus. Furthermore, the effects of Esterol10^®^ on the accumulation of HepG2 lipids were confirmed by determining the TG total content, both quantitatively and qualitatively, using the Oil Red stain and the lipid accumulation test, respectively, after 1 µM of Esterol10^®^, 1 µM RYR, and 1.25 µM of Atorvastatin for 24 h. As shown in Figure 6B, the total lipid accumulation determined by Oil Red staining was significantly higher (*p* < 0.05) in control cells than in other treatments; in particular, the attenuation of lipid accumulation was further contrasted by Esterol10^®^ compared to Atorvastatin and, even more evidently, by 1 µM RYR. Furthermore, Esterol10^®^ significantly reduced (*p* < 0.05) lipid levels compared to the control at 1 µM RYR and Atorvastatin (approximately 75%, 40%, and 88%, respectively). These results also demonstrate for the first time that Esterol10^®^ regulates lipid metabolism without side effects.

## 3. Discussion

In recent years, attention has been growing on possible preventive/alternative ways to avoid diseases, especially with regard to chronic diseases. Nutraceuticals have been proposed as key tools for the prevention and treatment of various conditions such as metabolic syndrome which includes hypercholesterolemia and cardiovascular disease [28]. In this context, the use of a dietary therapy supplement, associated with lifestyle improvement is the main alternative strategy to drug therapy to reduce serum LDL cholesterol [29]. Several meta-analyses and clinical studies have shown the correlation between decreased LDL levels and decreased relative risk of CVD, as explained by the direct relationship between a 1% reduction in LDL levels and a reduction >1% relative risk of CVD events [30]. The main advantage of supplements is that they are commonly used around the world, both in combination and as a substitute for prescribed medications as patients assume these products are safe [31]. For this reason, in recent decades, several studies have analyzed the effects of plant extracts with anti-cholesterol activity, but without considering the long-term side effects typical of statin treatments. RYR is widely used in prescriptions, as well as in alternative medicine and food supplements, in Asia, the United States, and European countries for its many biological properties including hypolipidemic and anti-atherosclerotic activities [32]. The results obtained from several studies have revealed that RYR is composed of more than 101 ingredients including monacolins and pigments which are the main component needed to explain its beneficial effects [32]. However, several safety, quality, and efficacy gaps have been reported, as stated by the FDA in 2013 [33] while the European Food Safety Authority (EFSA) has expressed its support for the use of RYR to keep plasma LDL levels low [34]. On the other hand, EFSA itself has not recently ruled out the possibility of risks related to the same dosages of monacolin K and argues that nutraceuticals containing a low level of RYR, can support the maintenance of low-intermediate CVD risk [35]. These findings are important to prevent the statin-like adverse events which occur in frail patients or in patients treated with RYR extracts at high content of monacolin K associated with drugs [2]. Recently, nutraceutical and plant extracts have been studied to explore new plant-based strategies well known in traditional medicine. The data on the efficacy of preparations of natural origin depends on the type of extract and the extraction conditions as reported for resveratrol or sage extracts [16,36]. However, the beneficial effects of natural extracts, for example, sage, resveratrol, or quercetin, have frequently been attributed to the antioxidant capacity related to the modulation of different molecular pathways involved in cell proliferation, survival, and metabolism. In this context, this is the first study that investigates the anti-cholesterol activity of a new formulation, called Esterol10^®^, obtained by combining Salvia Extract 10 µM, Quercetin 10 µM, Vitamin D3 1 nM, Vitamin K2 1 µM, CoQ10 1 µM, Resveratrol 1 µM, 9 nM astaxanthin, 1 µM sodium selenite, 1 µM folic acid and 1 µM RYR in an in vitro study. Indeed, when mixed together, the three extracts proved to be more effective than RYR in promoting the lowering of cholesterol in hepatocytes, through a reduction in the synthesis of cholesterol and its conversion into bile acids through the choleresis process which is a complex biochemical process that leads to the production of bile, an iso-osmotic electrolyte solution that forms in the liver as a product of its secretory function [24]. Taken together, these results demonstrated that Esterol10^®^ significantly improves hepatic metabolism by regulating the synthesis of cholesterol through the improvement of HMGCoA reductase and the reduction of its receptor expression. In this context, liver function is determined by the synthesis of total cholesterol while improving the production of bile acid and free cholesterol, as determined by tests for total cholesterol, free cholesterol, and bile acids. In fact, experimental results confirmed better anti-cholesterol efficacy when combined in a single blend, compared to RYR alone or Atorvastatin, suggesting the potential use of this formulation to supplement or replace statins. Anti-cholesterol therapies based on new and safer plant extracts and their mixtures are necessary to assist or replace statins. Although our results from in vitro experiments cannot be directly extrapolated to the antilipidemic and anti-cholesterolemic clinical effects, they must be confirmed in a more complex system, such as an in vivo model. At the same time, this study confirmed the molecular mechanism activated by Esterol10^®^ including AMPK, which has a direct impact on glucose and lipid metabolism [9]. Furthermore, the analysis showed that the formulation modulates SREBP, HMGCR, LDLr, and all other enzymes involved in cholesterol biosynthesis. In the present study, Esterol10^®^ is able to regulate cholesterol homeostasis by reducing PCSK9 and increasing the absorption of LDLr and LDL. The mixture of active ingredients used in this study is likely able to exert a more evident synergistic effect, reducing the cholesterol content and preventing liver damage.

## 4. Materials and Methods

### 4.1. Cell Culture

The human epithelial hepatocellular carcinoma HepG2 cells were purchased from the American Type Culture Collection (ATCC, Manassas, VA, USA) and were grown in Dulbecco’s modified Eagle’s medium (DMEM, Merck Life Science, Milano, Italy), supplemented with 10% fetal bovine serum (FBS, Merck Life Science, Milano, Italy), 2 mM L-glutamine (Merck Life Science, Milano, Italy) and 1% penicillin-streptomycin (Merck Life Science, Milano, Italy) at 37 °C and 5% CO_2_ [37]. The cells used in these experiments had a passage range of 90–95% [24]. After reaching 80–90% confluence, the cells were cultured in different ways based on different experimental protocols: 1 × 10^4^ cells in 96 well plates to study cell viability by MTT test and LDL uptake; 1 × 10^6^ cells were cultured in 6-well plates to measure the total cholesterol, and ALT quantification, HMGCoA reductase and related metabolic pathways using ELISA assay and Western blot analysis.

### 4.2. Experimental Protocol

HepG2 cells were used to verify the mechanism of action of several natural extracts, alone and combined, compared to RYR, in order to evaluate a possible new strategy to reduce cholesterol accumulation. Before stimulations, the cells were synchronized overnight with DMEM without red phenol and FBS, supplemented with 1% penicillin/streptomycin, 2 mM l-glutamine, and 1 mM sodium pyruvate in an incubator at 37 °C, 5% CO_2_, 95% humidity. During all the experiments, the medium was changed with a fresh medium with a high concentration of glucose (30 mM, Merck Life Science, Milano, Italy), and the untreated cells were grown in the presence of normal or 30 mM glucose as the controls [37]. Firstly, to evaluate the effects on HepG2 cells leading to finding the non-toxic concentration, a dose–response study was performed, testing the extracts in a range reported in the literature. Indeed, the cells were treated with increasing concentrations of sage extract (from 0.029 to 0.3 µM) [12], Red Yeast Rice (RYR, from 1 to 50 µM) [10], Vitamin D3 (from 1 to 100 nM) [38], Resveratrol (from 1 to 40 µM) [39], Quercetin (from 5 to 10 µM) [40], Astaxanthin (from 1.7 to 17 nM) [41], CoQ10 (from 1 to 100 µM) [42], Vitamin K2 (from 0.01 to 1 µM) [43], Sodium Selenite (from 1 to 10 µM) [44], Folic Acid (from 25 to 75 µg/mL) [45]. The best concentration of each extract was maintained for all successive experiments.

Secondly, the cells were stimulated with sage extract, Red Yeast Rice, Vitamin D3, Resveratrol, Quercetin, Astaxanthin, CoQ10, Vitamin K2, Sodium Selenite and Folic Acid, alone and combined (named Esterol10^®^) comparing data to 1 µM RYR alone [46] for 24 h, to exclude any cytotoxic effects by MTT test.

All substances, donated by Uriach Italy srl, were prepared directly in the simulation’s medium. In addition, total cholesterol, LDL concentration, and ALT activity were also analyzed using a colorimetric assay in order to evaluate cholesterol metabolism and the possible liver injury. Finally, additional experiments were carried out to verify the effectiveness of the combination (Esterol10^®^) compared to RYR alone analyzing HMGCoA reductase and the main intracellular pathways involved in cholesterol metabolism. The stimulation time was 24 h [9,47], corresponding to the duration of anticholesterolemic activity experiments as described above. Atorvastatin (1.25 μM), a concentration comparable with that available in the literature, was applied as a positive control [24] because it is a well-known anticholesterolemic substance [24]. All reported data were obtained by comparing results with those obtained on untreated cells under physiological or high glucose medium.

### 4.3. MTT Viability

A3-(4,5-Dimethylthiazol-2-yl)-2,5-Diphenyltetrazolium Bromide (MTT) test was performed following a standard protocol [48] to exclude cytotoxic effects. Briefly, after stimulations, both cell types were incubated with 1% of MTT dye (Merck Life Science, Milano, Italy) in DMEM white for 2 h at 37 °C in an incubator, and then purple formazan crystals were dissolved in an equal volume of MTT solubilization solution. Cell viability was determined by measuring the absorbance at 570 nm with correction at 690 nm, through a spectrometer (VICTOR × 4 Multilabel Plate Reader, PerkinElmer, Waltham, MA, USA), and calculated by comparing results to control cells without any stimulus (baseline 0%).

### 4.4. Measurement of Total and Free Cholesterol

The anticholesterolemic activity was determined by a Cholesterol Quantitation Kit (Merck Life Science, Milan, Italy) following the manufacturer’s instructions. The fractions of the total, free, and esterified cholesterol were analyzed [24]. Briefly, after stimulation, the medium was removed and the cells were extracted with 200 µL of chloroform:isopropanol:IGEPAL CA-630 (7:11:0.1). The samples were centrifuged at 13,000× *g* for 10 min and the supernatants were transferred to a new tube which was aired dry at 50 °C for 30 min to remove chloroform. Dried lipids were dissolved with 200 µL of the Cholesterol Assay Buffer with 50 µL of the Reaction Mix and the samples were incubated for 60 min at 37 °C protected from light. The absorbance was measured at 570 nm by a spectrometer (VICTORX4, multilabel plate reader) and the results were normalized to a sample protein concentration and expressed as µg/μL.

### 4.5. LDL Uptake Quantification

The LDL assay was performed using LDLC colorimetric assay kits (cholesterol oxidase/phenol aminophenazone method, Elabscience, Wuhan, China) according to the user manual [49]. Briefly, 2.5 µL of each sample was added to 180 µL of Reagent 1 and then incubated for 5 min at 37 °C. The absorbance was measured by a spectrometer at 546 nm (VICTOR × 4, multilabel plate reader) and the results were calculated by comparing data to control cells (baseline 0%).

### 4.6. The Bile Acids Production

The evaluation of the production of bile acids by the HepG2 cells, after stimulations were verified using the fluorometric assay (total bile acid assay) as reported in the literature [50]. Briefly, after stimulations, the cells were washed with cold phosphate-buffered saline (PBS), lysed by sonication in cold PBS 1X (Merck Life Science, Milan, Italy), and centrifuged at 10,000× *g* for 10 min at 4 °C. Bile acid determination is based on the fluorescence of resorufin measured by a spectrofluorometer at an excitation wavelength of 560 nm and an emission wavelength of 590 nm. The obtained results were normalized on the protein concentration and expressed as a percentage (%) compared to the control (0% line).

### 4.7. HMGCoA Reductase ELISA Kit

The HMGCoA Reductase ELISA Kit is suitable for the quantitative detection of HMGCoA Reductase/HMGCR (LSBio, Seattle, DC, USA) following the manufacturer’s instructions [51]. Briefly, cells after stimulations were collected, lysed by freezing and thawing 3 times, and centrifuged at 1500× *g* for 10 min at 2–8 °C. A volume of 100 µL of each sample was incubated with 100 μL of 1× Biotinylated Detection Antibody working solution for 60 min at 37 °C and then with 100 μL 1× HRP-Streptavidin Conjugate for 30 min at 37 °C. After that, 90 μL of TMB Substrate for 15 min at 37 °C in the dark and then 50 μL of Stop Solution were added to each well. The absorbance was measured at 450 nm using a plate reader (VICTORX4 multilabel plate reader) and the results were generated to a standard curve (0.625–40 ng/mL) and expressed as ng/μL.

### 4.8. Transaminase Analysis

The ALT assay (Merck Life Science, Milan, Italy) was performed using a colorimetric method to evaluate the levels and activity of metabolic enzymes which are elevated in damaged tissues especially in liver tissue, according to the manufacturer’s recommendations [52]. Briefly, the cells were homogenized with 200 µL of ALT Assay Buffer and then centrifuged at 15,000× *g* for 10 min. A volume of 100 µL of the Master Reaction Mix was added to each sample and after 2–3 min, the measurement was started at 570 nm using a plate reader (VICTORX4 multilabel plate reader). The plate protected from light was incubated at 37 °C taking measurements every 5 min. The final measurement was the penultimate reading before the most active sample was near the end of the linear range of the standard curve. The results were generated comparing data to the standard curve (0–10 nmol/mL) and expressed as milliunit/mL.

### 4.9. Lipid Accumulation Assay

Total lipid accumulation was evaluated by Hepatic Lipid Accumulation Assay Kit (Abcam, Cambridge, UK) following the recommendations of the instructions [37]. Briefly, after stimulation, the cells were fixed in 75 μL of Fixative for 15 min, washed with 100 μL of Wash Solution, stained with 75 μL of Oil Red O Working Solution for 20 min at room temperature, and observed under a microscope (Leica DM1000). In order to quantify the lipid accumulation, 100 μL of Dye Extraction Solution were added to each well, gently mixed for 25 min, and the absorbance was measured at 490–520 nm using a plate reader (VICTORX4 multilabel plate reader). The results were obtained comparing samples OD to control cells (baseline 0%).

### 4.10. SREBP-2 Detection Assay

At the end of stimulations, cells were lysed to evaluate the levels of SREPBP-2 following the ELISA kit manufacturer’s instruction (LSBio, Seattle, DC, USA) [51]. A volume of 100 μL of samples was added to 96 well ELISA plates and incubated for 2 h at 37 °C. After that, 100 μL of Detection Reagent A was added to each well for 60 min at 37 °C in agitation. Then, the plate was incubated with 100 μL of Detection Reagent B for 60 min at 37 °C. At the end, 90 μL of TMB Substrate solution was added for 15 min at 37 °C in the dark. Finally, 50 μL of Stop Solution was added to the plate and the absorbance was measured at 450 nm using a plate reader (VICTORX4 multilabel plate reader). The results were obtained comparing data to standard curves (0.312–20 ng/mL) and expressed as ng/μL.

### 4.11. ERK/MAPK Detection Assay

ERK/MAPK activities were measured by the InstantOne™ ELISA (Thermo Fisher) on cell lysates following the manufacturer’s instructions [53]. Briefly, cells at the end of treatments were lysed with 100 μL Cell Lysis Buffer Mix, 50 μL/well of each sample was tested in InstantOne ELISA microplate strips, and in each well, the Antibody Cocktail was added and incubated for 1 h at room temperature on a microplate shaker. At the end, the Detection Reagent was added to each well, and after 20 min, the reaction was stopped by adding a stop solution to each. The strips were measured by a spectrometer (VICTOR X4, multilabel plate reader) at 450 nm. The results were expressed as mean absorbance (%) compared to the control.

### 4.12. PCSK9 Detection Assay

PCSK9 activity was measured by analyzing PCSK9 level in culture supernatants of HepG2 cells, measured using an ELISA kit according to the manufacturer’s instructions (BioVision, CA, USA; Elabscience Biotechnology, Wuhan, China) [54]. Briefly, 100 µL of biotinylated anti-human PCSK9 antibody working solution was added into each well and the plate was incubated at 37 °C for 60 min; the wells were washed with PBS 1× and then incubated for an additional 30 min at 37 °C with 100 µL of ABC working solution. At the end of the time, 90 µL of TMB was added into each well and the plate was incubated at 37 °C in the dark for 20–25 min. Color development at 450 nm was measured using a microplate reader (VICTOR X4 Multilabel Plate). The results were calculated by generating a standard curve (0–10 pg/mL) and expressed as pg/mL.

### 4.13. Cell Lysates and Western Blot

HepG2 cells were lysed in ice with Complete Tablet Buffer (Roche, Basel, Switzerland) supplemented with 2 mM sodium orthovanadate (Na3VO4), 1 mM phenylmethanesulfonyl fluoride (PMSF) (Sigma-Aldrich, St. Louis, MO, USA), 1:50 mix Phosphatase Inhibitor Cocktail (Sigma-Aldrich, St. Louis, MO, USA), and 1:200 mix Protease Inhibitor Cocktail (Sigma-Aldrich, St. Louis, MO, USA). According to the standard protocol, 35 μg of protein of each sample was resolved on 10% SDS-PAGE gels, and polyvinylidene difluoride membranes (PVDF, GE, Healthcare Europe GmbH) was incubated overnight at 4 °C with the following specific primary antibodies: anti-LDL receptor (1:500; Santa Cruz, CA, USA), anti-pSRC (1:500; Santa Cruz, CA, USA), anti-SRC (1:500; Santa Cruz, CA, USA), anti-pAMPK (1:500; Santa Cruz, CA, USA), anti-AMPK (1:500; Santa Cruz, CA, USA) and anti-HMGCr (1:500; Santa Cruz, CA, USA). Protein expression was normalized and verified through anti-β-actin (Sigma-Aldrich, St. Louis, MO, USA). The results were expressed as means ± SD (% vs. control).

### 4.14. Statistical Analysis

Data reported were obtained from at least five independent experiments performed in triplicate for each experimental protocol and analyzed using Prism GraphPad statistical software. Results reported are expressed as means ± SD using a one-way ANOVA followed by Bonferroni post hoc test for statistical analysis. *p* values < 0.05 were considered statistically significant.

## 5. Conclusions

Based on current results, Esterol10^®^ may hold promise as a novel cholesterol-lowering approach with a statin-like but naturally derived mechanism of action [5] with fewer side effects. These natural extracts, containing antioxidants, or in any case able to counteract oxidative stress, can represent a promising adjuvant therapy in hypercholesterolemia, reducing the risk of cardiovascular diseases by increasing the resistance to oxidation of circulating lipids [8]. In conclusion, this study demonstrates for the first time that the combination of 10 µM Sage Extract, 10 µM Quercetin, 1 µM Vitamin D3, 1 µM Vitamin K2, 1 µM CoQ10, 1 µM Resveratrol, 9 nM Astaxanthin, 1 µM Sodium Selenite, 1 µM Folic Acid, and 1 µM RYR, called Esterol10^®^, can be a candidate to be an effective treatment to avoid the accumulation of cholesterol, being also capable of modulating the hepatic metabolism. Furthermore, these results confirm that the combination of active ingredients could be a potential strategy, as the curative effects are enhanced, reducing the risk of adverse effects of the individual components. This complementary therapy could therefore be combined with classic statins to improve success in reducing dyslipidemia. In addition, the results provide new insights about the molecular mechanism of hypocholesterolemia, strongly supporting the employment of Esterol10^®^ as a functional food able to act as a potential hypolipidemic agent without adverse effects. However, further experiments will be planned in the future to confirm data in vitro on an in vivo model before testing it in humans.

## Data Availability

Raw data are preferably deposited at the Laboratory of Physiology (C. Molinari), ensuring appropriate measures so that raw data are retained in full forever under a secure system. The data presented in this study are available on reasonable request from the corresponding author.

## Abbreviations

ALT	alanine aminotransferase
AMPK	AMP-activated protein kinase
CHD	coronary heart disease
CVD	cardiovascular
DMEM	Dulbecco’s modified Eagle’s medium
EFSA	European Food Safety Authority
ERK	extracellular signal-regulated kinases
FBS	fetal bovine serum
FDA	US Food and Drug Administration
GS/GSSG	reduced glutathione/oxidized glutathione
HDL	high-density lipoprotein
HDL-C	high-density lipoprotein-associated cholesterol
HMGCoA	3-hydroxy-3-methyl-glutaryl-CoA
HMGCR	3-hydroxy-3-methyl-glutaryl-coenzyme A reductase
LDL-C	low-density lipoprotein-associated cholesterol
LDLr	LDL receptor
MTT	3-(4,5-Dimethylthiazol-2-yl)-2,5-Diphenyltetrazolium Bromide
PBS	phosphate-buffered saline
PCSK9	proprotein convertase subtilisin/kexin type 9
PMSF	phenylmethanesulfonyl fluoride
PVDF	polyvinylidene difluoride
RYR	Red Yeast Rice
SREBP	sterol responsive element binding protein
TG	triglycerides
VLDL	very-low density lipoprotein
WHO	World Health Organization

## Appendix A

**Table A1:** Original data of HMGCoA, PSK9, SREBP-2, and ERK/MAPK activity on HepG2 cells. RYR = Red Yeast Rice. Data are mean ± SD of five independent experiments performed in triplicate.

	HMGCoA Reductase	PCSK9	SREBP-2	ERK/MAPK
Control	1.2 ± 0.27	3123 ± 0.38	2279 ± 0.24	3383 ± 0.28
Esterol 10^®^	1.75 ± 0.33	5009 ± 0.21	1026 ± 0.29	5412 ± 0.33
RYR 1 µM	1.56 ± 0.42	4751 ± 0.26	1436 ± 0.34	4905 ± 0.42
Atorvastatin	1.43 ± 0.45	3840 ± 0.29	1687 ± 0.27	4228 ± 0.31
